# Non-classical crystallization of CeO_2_ by means of *in situ* electron microscopy†

**DOI:** 10.1039/d3nr02400h

**Published:** 2023-08-23

**Authors:** Hannes Zschiesche, Inna L. Soroka, Mats Jonsson, Nadezda V. Tarakina

**Affiliations:** a Max Planck Institute of Colloids and Interfaces, Department of Colloid Chemistry Potsdam Germany Hannes.Zschiesche@mpikg.mpg.de Nadja.Tarakina@mpikg.mpg.de; b Applied Physical Chemistry, School of Engineering Sciences in Chemistry, Biotechnology and Health, KTH Royal Institute of Technology Stockholm Sweden

## Abstract

During *in situ* liquid-phase electron microscopy (LP-EM) observations, the application of different irradiation dose rates may considerably alter the chemistry of the studied solution and influence processes, in particular growth pathways. While many processes have been studied using LP-EM in the last decade, the extent of the influence of the electron beam is not always understood and comparisons with corresponding bulk experiments are lacking. Here, we employ the radiolytic oxidation of Ce^3+^ in aqueous solution as a model reaction for the *in situ* LP-EM study of the formation of CeO_2_ particles. We compare our findings to the results from our previous study where a larger volume of Ce^3+^ precursor solution was subjected to γ-irradiation. We systematically analyze the effects of the applied irradiation dose rates and the induced diffusion of Ce ions on the growth mechanisms and the morphology of ceria particles. Our results show that an eight orders of magnitude higher dose rate applied during homogeneous electron-radiation in LP-EM compared to the dose rate using gamma-radiation does not affect the CeO_2_ particle growth pathway despite the significant higher Ce^3+^ to Ce^4+^ oxidation rate. Moreover, in both cases highly ordered structures (mesocrystals) are formed. This finding is explained by the stepwise formation of ceria particles *via* an intermediate phase, a signature of non-classical crystallization. Furthermore, when irradiation is applied locally using LP scanning transmission electron microscopy (LP-STEM), the higher conversion rate induces Ce-ion concentration gradients affecting the CeO_2_ growth. The appearance of branched morphologies is associated with the change to diffusion limited growth.

## Abstract 2

The approach of comparing *in situ* liquid-phase electron microscopy study of ceria formation to results from gamma radiolysis covers the influence of orders of magnitude different dose rates and adjustable irradiation on non-classical crystallization.

## Graphical abstract

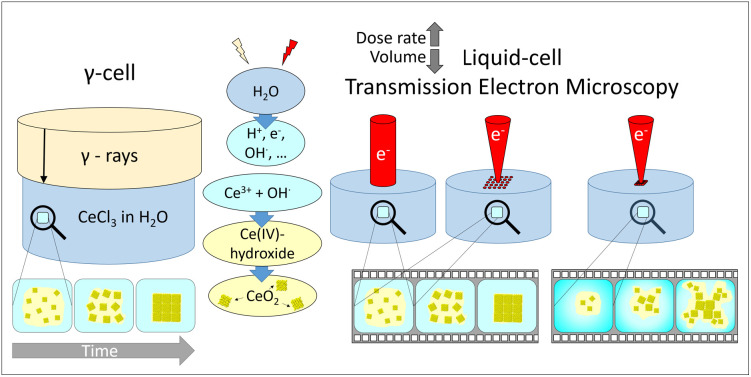

Radiation chemistry in aqueous solutions has been demonstrated to be a versatile method for tailored synthesis of nanomaterials.^1–6^ Upon exposure to ionizing radiation, such as high-energy electrons or gamma photons, water undergoes radiolysis producing oxidants (HO˙, HO_2_˙, H_2_O_2_ and O_2_) as well as reductants (e_aq_^−^, H˙ and H_2_).^7,8^ The radical species formed upon radiolysis in general display high reactivity towards most solutes. The rate of radiolytic production of a specific species is given by the dose rate (depending on the activity or intensity of the radiation source) and the corresponding radiation chemical yield (*G*-value).^9,10^ The *G*-values depend on the type and the energy of the radiation. For gamma photons and high-energy electrons, the main products are HO˙ and e_aq_^−^, with *G*-values of 0.28 and 0.27 μmol J^−1^, respectively. Depending on the type of ionic solution and the targeted nanomaterial, the redox conditions can be optimized using radical scavengers. For the synthesis of a nanomaterial utilizing an oxidizing hydroxyl radical (HO˙), the most convenient scavenger of the hydrated electron (e_aq_^−^) is N_2_O, which upon reaction with e_aq_^−^ produces HO˙ in aqueous solution. Hence, by saturating or continuously purging an aqueous solution with N_2_O, the rate of hydroxyl radical production is almost doubled, while the possible interference from the strongly reducing hydrated electron on the nanomaterial synthesis is efficiently minimized. When reducing radicals are required to drive the synthesis (*e.g.*, when synthesizing metal nanoparticles from metal ions in solution), hydroxyl radical scavengers are used. The most common scavengers for this purpose are 2-propanol and formate.^11^

A fairly recent example of radiation induced synthesis of a nanomaterial utilizing the oxidative route is the production of CeO_2_ from CeCl_3_ aqueous solution.^12^ In this process, hydroxyl radicals formed upon radiolysis of water, oxidize highly soluble Ce^3+^ to sparsely soluble Ce^4+^. The latter forms hydrated Ce(iv) hydroxides, which serve as intermediates in the liquid-to-solid phase transformation. The primary CeO_2_ particles nucleate and grow (up to 3 nm in diameter) inside the intermediate gel-like phase. Thereafter, they mutually align, guided by the confinement of this phase, forming hierarchical structures, mesocrystals.^13,14^ It is worth mentioning that through the current manuscript, we will refer to the definition of the primary particles as nanoparticulate building blocks from which larger particles, aggregates, are formed. In the above mentioned study the aqueous solution of CeCl_3_ was saturated with N_2_O prior to irradiation. No other additives were used to control the growth of the particles. As the dose rate of the gamma source was relatively low (*ca.* 0.1 Gy s^−1^), the time required to reach a significant conversion of the Ce^3+^ to Ce^4+^ was substantial (of about 20 hours). It was therefore possible to isolate samples from the solution at different stages of conversion and analyze them by *ex situ* electron microscopy (EM). Based on these measurements, a mechanism for CeO_2_ mesocrystal formation was proposed.

Admittedly, this way of monitoring the reaction has certain drawbacks since all the samples are analyzed after evaporation of the solvent, which makes it difficult to confirm the exact speciation in solution, and avoid drying artifacts. A way to circumvent the problem of solvent evaporation is to use *in situ* liquid-phase electron microscopy (LP-EM)^15^ where the ionizing radiation from the electron beam in the electron microscope drive the radiolysis of the water contained in the liquid-cell (LC) sealed against vacuum.^16^ This opens up the possibility to directly investigate the primary particle formation and the subsequent phase formation in real time.^17–23^ In addition, the possibility to easily adjust dose rates (by varying the irradiation conditions) is another advantage of LP-EM, which is assumed to influence the conditions for the nucleation and growth of particles.^24,25^

Note, that the irradiation conditions for conventional Gamma-Cell and for LP-EM are quite different. In fact, the dose rates differ by at least six orders of magnitude. As it was demonstrated previously, metal nanoparticle formation, driven by radiolytic reduction, is strongly dependent on irradiation dose rates.^26^ Therefore, the results of the current study (using LP-EM) may differ significantly from the results of previous experiments on CeO_2_ formation conducted under gamma radiolysis. Furthermore, the extremely high dose rates during LP-EM exposures will lead to rapid conversion of the dissolved species in the irradiated volume to less soluble ones, followed by sequential formation of the solid phase. Consequently, this may provoke diffusion of non-reacted ions into the irradiated volume from the surrounding non-irradiated volume.^16^ The diffusion will lead to inhomogeneous reaction conditions that affect the process of crystal growth.

In this work we explore a non-classical crystallization route using *in situ* LP-EM. We choose the formation of CeO_2_ mesocrystals as a model process because of several reasons: (1) it allows us to use a water solution of a Ce^3+^ salt without any organic surfactants/additives; (2) radiolytic oxidation has been relatively little studied both in a LP-EM and upon gamma-radiation, and the formation mechanism in LP-EM conditions is not clear; (3) we compare a radiolytic process in a LP-EM confined cell and in a large volume of a Gamma-Cell^12^ thus enabling a unique study of the influence of the irradiation dose rate on nucleation and crystal growth by combining bulk and nanoscale approaches. Furthermore, we take advantage of the versatile irradiation conditions provided in LP-EM to study the influence of induced concentration gradients on the crystal growth during the radiolytic oxidation in solution.

## *In situ* formation of CeO_2_ mesocrystals by LP-EM applying homogeneous electron beam irradiation

**Fig. 1:**
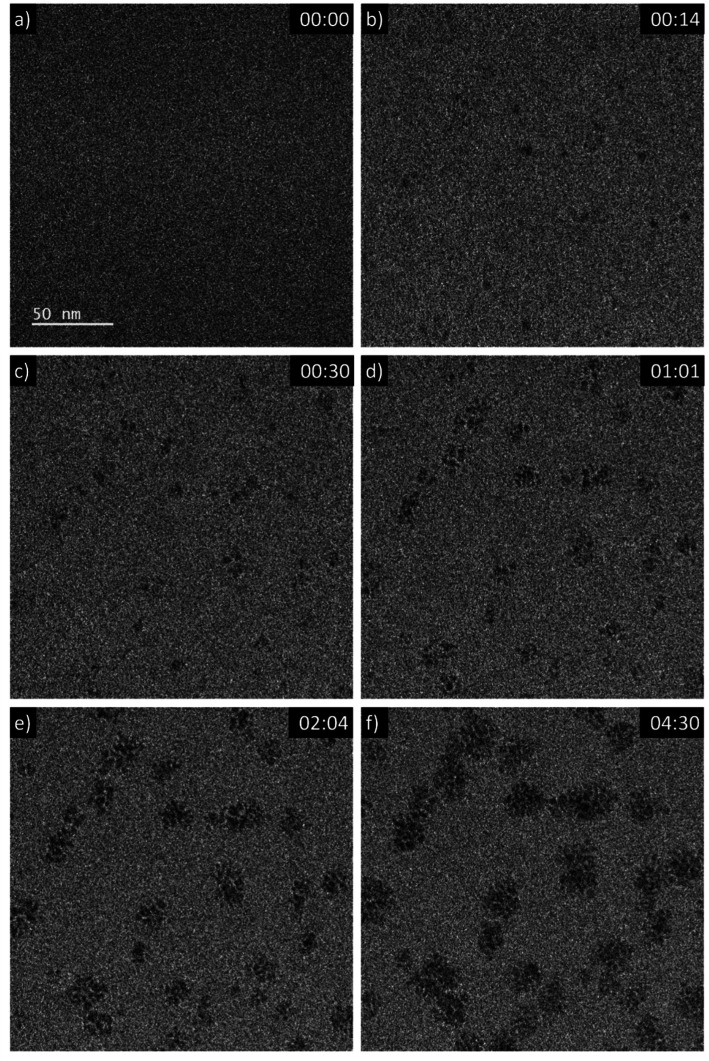
Sequential images from an *in situ* LP-TEM movie showing the formation of aggregates (see video “V01_LP-TEM_CeO_2_-nanoparticles.avi”, time given as mm:ss). A dose rate of 16.7 × 10^6^ Gy s^−1^ is applied.

**Fig. 2:**
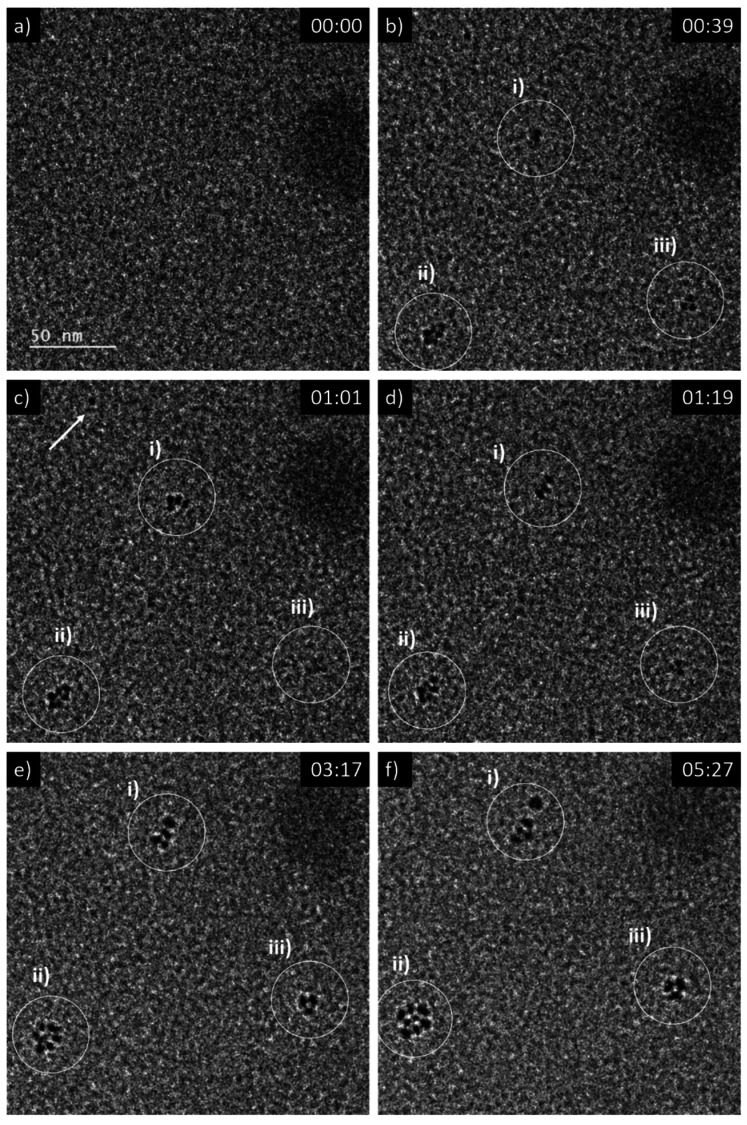
Sequential images from an *in situ* LP-TEM movie showing (a)–(b) formation and (c)–(f) arrangement of primary particles when a dose rate of 16.7 × 10^6^ Gy s^−1^ is applied. Primary CeO_2_ particles can be resolved and evaluated quantitatively, because of lower thickness of the liquid in comparison to Fig. 1. (See also according video “V02_LP-TEM_CeO_2_-nanoparticles”; time given as mm:ss).

A 5 mM aqueous solution of CeCl_3_·7H_2_O was purged with N_2_O prior to liquid phase transmission electron microscopy (LP-TEM) investigations. Immediately after applying a homogeneous irradiation by the electron beam (dose rate of 16.7 × 10^6^ Gy s^−1^, see ESI† for dose rate estimation) CeO_2_ primary particles are formed and aggregated (Fig. 1). The aggregates grow upon continuous irradiation of solution. Fig. 1 presents images from a TEM LC with a thick layer of liquid in which many aggregates form. However, the achieved spatial resolution is insufficient to follow in detail the formation and growth of primary particles. Therefore, another experiment with a LC that contains a thinner layer of liquid is performed. The latter enables us to reach higher spatial resolution and thus, to track the development of primary particles (Fig. 2). The average diameter of primary particles develops from 3.9 ± 0.5 nm to 5.3 ± 1.2 nm (Fig. S1†), while the size of aggregates converges to approximately 25 nm (Fig. S1†).

**Fig. 3:**
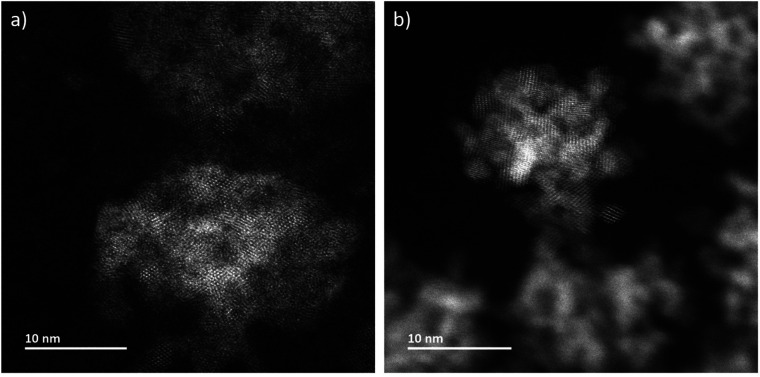
ADF-STEM images of CeO_2_ grown (a) in LP-TEM (dose rate 16.7 × 10^6^ Gy s^−1^) and (b) under steady-state gamma radiolysis.

In addition to *in situ* characterizations, LCs have been opened at the end of each experimental session and have been dried at environmental conditions in order to investigate them *ex situ* without superimposing signals from liquid and one of the two LC-membranes. Atomically resolved annular dark-field scanning transmission electron microscopy (ADF-STEM) imaging determines that the aggregates are composed by nanocrystals (Fig. 3a and S2†). The microstructure of CeO_2_ formed during LP-TEM investigations strongly resembles the CeO_2_ mesocrystals grown by gamma-radiation (Fig. 3b).^12^ Moreover, the primary particles are found to mutually align in highly ordered structures (see Fourier transformation in Fig. S2†). Finally, energy dispersive X-ray spectroscopy (EDS) confirms the components of CeO_2_ (Fig. S3†).

## Radiolytic formation of CeO_2_ primary particles *versus* metal nanoparticles

The size of the primary CeO_2_ nanoparticles synthesized from 5 mM cerium precursor solution using gamma-radiation induced synthesis was found to be ∼3 nm, which is very close to what is found under the LP-TEM irradiation (previous section, Fig. S1† and Fig. 3b in ref. 12). This is quite surprising given the fact that for radiation synthesis of metal nanoparticles, the particle size displays strong dose rate dependence.^11,27^ The rationale for the latter is that the concentration of nucleation sites increases with increasing dose rate, which in turn leads to formation of more and smaller particles when those are immediately stabilized by surfactants against coalescence or other merging processes.

For CeO_2_ synthesis, a dose rate difference by eight orders of magnitude does not have any considerable impact on the size of the primary particles. Thus, the rate-limiting step of the nucleation process must be different for CeO_2_ compared to metal nanoparticles and appears independent from the dose rate of the ionizing radiation in the studied range, and therefore of radical concentration. This is very well in line with the mechanism proposed for gamma-radiation induced synthesis of CeO_2_ from Ce^3+^ in aqueous solution.^12^ In this mechanism, nucleation takes place in a gel-like intermediate phase consisting of hydrated Ce(iv)-hydroxides formed after the radiolytic oxidation of Ce^3+^. This subsequent nucleation, followed by growth of CeO_2_ inside the intermediates, is not driven by radicals and, therefore, depend to a lesser extent on the irradiation dose rate.

## Primary CeO_2_ particle reorganization towards mesocrystals

Mutual alignment of the resolved primary CeO_2_ particles can be recognized in the abovementioned observations (Fig. 1 and 2) and from *ex situ* structural and compositional analysis after opening the TEM LC (Fig. 3 and S2†). This mutual alignment of the primary particles is generally in agreement with previously presented *ex situ* interpretations.^12^ In the current study, the relative orientations of primary particle in the aggregates (i), (ii) and (iii) in Fig. 2 (or in the video “V02_LP-TEM_CeO_2_-nanoparticles”) change, even though those particles are already connected to each other. Particle–particle interactions supposed to be present and may be described either by van der Waals forces, polarity forces, *etc.*, or by the formation of atomic solid–solid interfaces.^28^ If primary CeO_2_ particles connect through a solid–solid interface formation, a reorganization may take place by deformation within the primary particle instead of a respective reorientation of the entire particle.^29,30^ If long-range interactions (*e.g.* van der Waals forces) are established, mutual alignment, during which entire primary particle moves, may take place. The aggregates may be kept together by a constrained environment (gel-like Ce^IV^-hydroxide phase) in this case, as previously proposed.^12^ Atomic resolution is not available within the used LC configuration *in situ*. Thus, no atomic solid–solid interface formation could be clearly stated between primary CeO_2_ particles. Also, no gel-like constraining phase could be identified, because of limited contrast in LP-TEM imaging. However, single primary particles appear to be organized as a whole (Fig. 1 and 2) indicating that the latter description applies for the CeO_2_ mesocrystal formation.

## Comparison of CeO_2_ aggregates growth in LP-TEM and low dose rate steady-state gamma radiolysis

The CeO_2_ reaction pathway in LP-TEM appears to be not directly influenced by the dose rate. However, the higher concentration of radicals enables a faster consumption of Ce^3+^. This becomes obvious comparing the time difference of the mesocrystal formation under low dose rate steady-state gamma radiolysis (hours) and mesocrystal-like aggregates in LP-TEM (minutes). Nevertheless, the size saturation of aggregates obtained under steady-state gamma radiolysis and in LP-TEM (approximately 20–30 nm) is in the same order of magnitude at the stage when all available Ce^3+^ is expected to be consumed. Generally, the size of transformed intermediate gel-like phase in solution determines the size of the formed aggregates.^31,32^ Our result suggests that these sizes of aggregates are not influenced by the different applied dose rates and therefore by radical concentrations.

## Diffusion effects and formations of branched structures

**Fig. 4:**
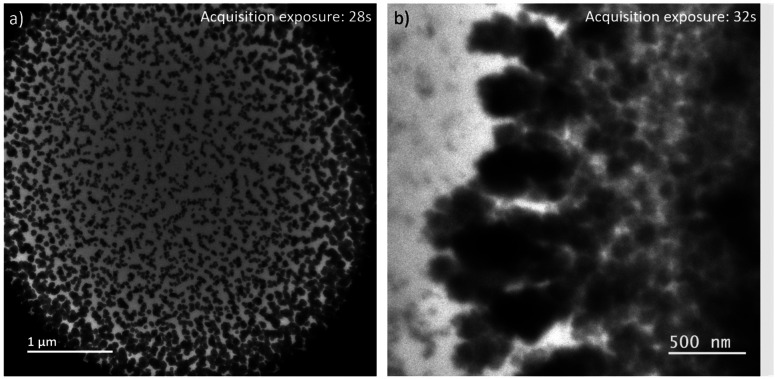
LP-TEM images of (a) full view on irradiated liquid volume after approximately 40 min of exposure. (b) Branched structures appear outside the previously irradiated volume.

By expanding the view from the central parts of the irradiated volumes in LP-TEM to their rims, aggregates of larger size are identified (see Fig. 4a). Differences in their size and shape can be caused by concentration gradients (Ce^3+^ ions) and explained as follows. The extreme irradiation conditions result in almost immediate depletion of the Ce^3+^ ions in the irradiated region. This depletion limits the growth of CeO_2_ mesocrystals as described above. At the same time, the depletion results in Ce^3+^ concentration gradients between the irradiated and non-irradiated volumes of solutions. This concentration gradient leads to the diffusion of Ce^3+^ into the rim of the irradiated volume. That diffusion acts as additional Ce^3+^ supply. After entering the irradiated volume, radiolytic oxidation of Ce^3+^ can occur replenishing the existing or forming additional Ce(iv)-hydroxide phase in which CeO_2_ nucleation takes place. This process ultimately leads to additional crystal growth.^26,33^

Outside the irradiated volume, we observed formation of branched structures (Fig. 4b). Following the formation mechanism described above, Ce^3+^ ions cannot be oxidized outside the irradiated volume, because of a lack of radicals that are suspected to recombine faster than to diffuse outside the irradiated volume. That raises the general question, how CeO_2_ can grow into a non-irradiated volume. In general, the interface between the irradiated and non-irradiated volume has significantly different properties than either of these volumes. Assuming an atomically sharp interface between irradiated and non-irradiated volume, only a diffusion (random walk) of formed CeO_2_ particles or aggregates, or Ce(iv)-hydroxide could cause formation of branched CeO_2_ morphologies in the non-irradiated volume. Knowing that the investigated sample is a confined liquid of not negligible thickness, an idea of atomically sharp interface does not hold. Thus, there might be a zone outside the directly irradiated volume in which significantly less, but still enough high energy electrons are scattered into^34,35^ providing a significant concentration of radicals to initiated oxidation of Ce^3+^. The lower radical concentration in comparison to the directly irradiated volume causes a lower oxidation rate of Ce^3+^. The slower transformation process is thereby effected by an already established Ce^3+^ concentration gradient and diffusion between non-irradiated and irradiated volume. This may cause the observed branched morphology,^36^ because of additional, but most likely inhomogeneous Ce^3+^ supply. As soon as CeO_2_ has formed, secondary effects, such as multiple scattering of high energy electrons, can be further enhanced since CeO_2_ has a higher probability for scattering of those electrons than the liquid solution. Simulations would be needed to verify these suggestions or raising the necessity to find other explanations.

A more insightful experimental proof is limited by the fact that no *in situ* information about formations of such branched aggregates at the interface is available (no direct irradiation, no measureable signal for *in situ* LP-TEM data). However, one has to re-call that imaging by liquid phase scanning transmission electron microscopy (LP-STEM) provides spatially and temporally non-uniform irradiation by a small converged probe with a diameter in the order of a nanometer (or less). Thus, the growth of branched structures, as observed in LP-TEM at the rim of the irradiated volume, is expected to be inducible by LP-STEM intrinsically. The dose rate within the converged beam can be even up to five orders of magnitude higher than that in the described LP-TEM (see dose rate estimations in ESI†). The irradiation can be compared to a consecutive pulse exposure, but with fairly extreme doses per pulse (as compared to irradiation with a pulsed electron beam from an accelerator, *e.g.* ref. 37). This enables principally a systematic *in situ* study of the influence of irradiation condition on diffusion effects. The dose rate within the converged electron beam can be directly modified by a change of the current in the electron beam. Or, the scan step distance (spatial distance between pulses) can be changed, which modifies overlaps or distances between pulses and, thereby, the dose rate applied in average on an exposed volume.^38^

**Fig. 5:**
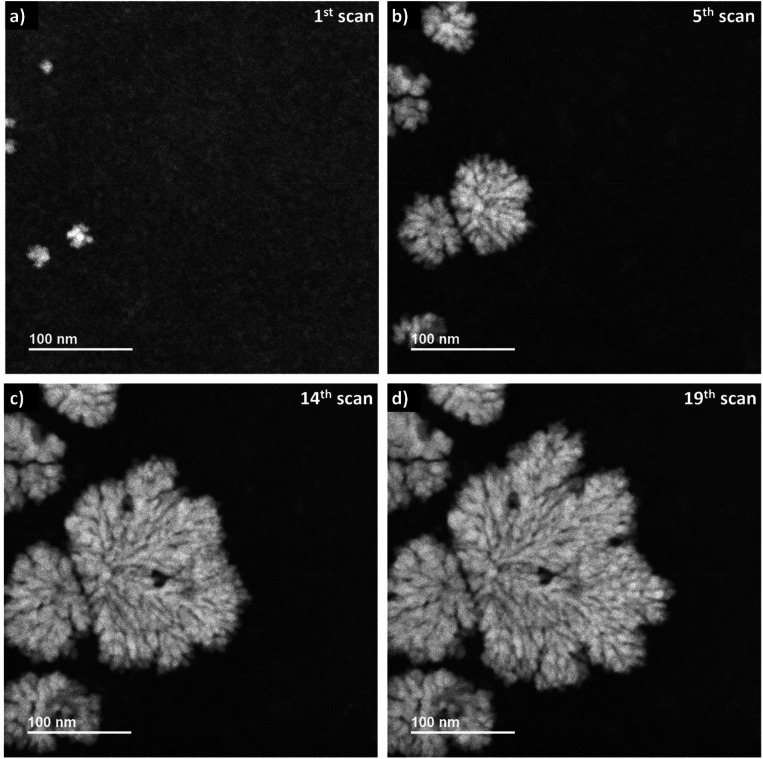
Sequential *in situ* ADF imaging in LP-STEM. An average dose rate of 5.9 × 10^6^ Gy s^−1^ is applied. (a) Already within the first scan, the aggregates become visible. (b) Continuous growth of aggregates while no formation of the additional ones is observed. (c) The appearance of the growing aggregates changes from isotropic to branched structures. (d) Further development of branched structures to larger sizes (video “V03_LP-STEM_CeO_2_-nanoparticles.avi”).

When applying a frame-averaged dose rate of 5.9 × 10^6^ Gy s^−1^ in LP-STEM, the aggregates form immediately during the first irradiation scan, (Fig. 5a). Their number does not change upon further irradiation (Fig. 5b). However, their initially isotropic appearance becomes anisotropic (Fig. 5c) and eventually, branched structures form (Fig. 5d). The average size of the formed branched structures is significantly larger than the sizes of the previously observed mesocrystal-like aggregates (Fig. S4†). Note, that a large uncertainty in size is given by the strong variation in size of the six grown branched structures. More importantly, the average size does not converge towards a constant value during the irradiation period. The branched structures grow continuously, although they have already reached sizes larger than the mesocrystals, formed and observed in LP-TEM. The formation of larger particles can again be attributed to additional supply of Ce^3+^ by diffusion effects.^26,33^ The diffusion effects (continuous supply of Ce^3+^ ions) are known to cause branched structures.^22^ Similar to our very initial comment on the origin of branched structure formation, diffusion limited aggregation of CeO_2_ particles could basically also describe the observed process.^39,40^ However, no individual CeO_2_ primary particles or aggregates are observed that merge to the growing branched structures in LP-STEM.

## Isotropic growth in LP-STEM at lower average dose rates

**Fig. 6:**
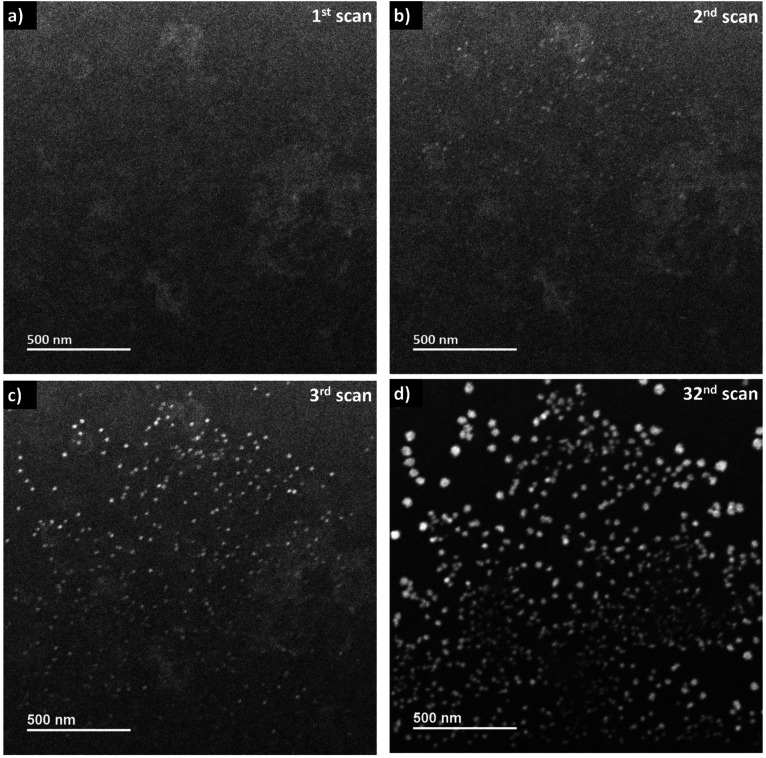
Sequential *in situ* ADF imaging in LP-STEM. An averaged dose rate of 2.4 × 10^5^ Gy s^−1^ is applied. (a) Initial scan. (b) Nano-sized aggregates become visible in the second scan and (c) their contrast increases during exposure. (d) The projected size of the aggregates increases constantly while almost no new ones are formed. (see video “V04_LP-STEM_CeO_2_-nanoparticles.avi”).

When the frame-averaged dose rate is reduced by one order of magnitude to 2.4 × 10^5^ Gy s^−1^ by expanding the scanning steps in LP-STEM, CeO_2_ aggregates form as well (Fig. 6). However, their sizes are significantly smaller than the sizes of branched structures (Fig. S5†). Furthermore, their appearance remains isotropic and does not evolve to branched structures with longer exposure (Fig. 6d). The spatial separation of pulses might reduce significantly the concentration gradients, and thereby diffusion effects.^16^ Potentially, conditions similar to those for uniform irradiation in LP-TEM are established. This statement is supported by the fact that the formed aggregates possess uniform size of about 30–40 nm, that is comparable to the size of mesocrystals (of about 25 nm) obtained in LP-TEM. However, the average size of aggregates appears to have not reached a constant value with increasing cumulative radiation dose (Fig. S5†). Thus, further Ce^3+^ diffusion from the outside of the irradiated liquid volume (also not previously irradiated) may still take place and effect the growth. The transition from the growth of branched structures to growth of spherical structures for lower dose rates in LP-STEM has been observed previously and described in the literature.^22^

Interestingly, while only one kind of aggregates (spherical or branched structures) are observed in the LP-STEM depending on the applied dose rate (Fig. S6†), aggregates of various morphology are formed simultaneously in LP-TEM (Fig. 4). The latter is argued by the presence of both, negligible diffusion effects on the growth in the central part of homogeneously irradiated liquid volume and significant diffusion effects at the rim of the irradiated liquid volume. In contrast, diffusion effects may always play a key role in LP-STEM and growth of spherical-like or branched structures can be adjusted in dependence of the in average applied dose rate.

## Conclusions

CeO_2_ nucleation and growth from aqueous CeCl_3_·7H_2_O solutions using ionizing electron irradiation have been studied using LP-EM. A direct comparison of experiments performed in LP-TEM and in a Gamma-Cell on identical material systems has been carried out. It is found that the orders of magnitudes higher dose rates in LP-TEM do not alter the CeO_2_ multistep crystallization pathway (typical for non-classical crystallization) established earlier using low dose rate steady-state gamma-radiation.

The conversion rate of Ce^3+^ to Ce^4+^ is affected by the higher dose rates. Consequently, the aggregates grow much faster during LP-TEM experiments than during gamma-radiation induced synthesis. However, the sizes of the formed primary CeO_2_ particles are close to each other, indicating a dose-rate-independent nucleation process of primary CeO_2_ particles for the investigated dose rate range.

Furthermore, a gradient in size of the formed CeO_2_ aggregates is determined from the rim of irradiated liquid volume to its center in LP-TEM. This is caused by diffusion of Ce^3+^ from the outside of irradiated liquid volume towards its center. Thus, an additional supply of Ce^3+^ can lead to continuous growth of the metal oxide aggregates and is therefore a parameter to tune their size.

Finally, we propose that depending on induced Ce^3+^ concentration gradients a change of the growth mode can be triggered from approximately spherical to complex branched nanostructure morphologies. The growth of those morphologies can be controlled and uniquely produced employing periodic pulsed irradiation in LP-STEM.

## Methods

In the LP-EM experiments 5 mM aqueous solution of CeCl_3_·7H_2_O (98.5% purity, Merck), saturated with N_2_O, were used. The solutions were load into a LC following Protochips’ standard routine.^41^ The used LC consists of Protochips’ “No spacer” bottom E-chip and Protochips’ “Microwell” 8 × 16 top E-chip. Protecting layers on the E-chips were removed by washing in acetone and methanol. Then, E-chips were gently dried by compressed air. Then, E-chips have been plasma cleaned in order to make their surfaces hydrophilic and facilitate spreading of the dropped liquid. A liquid volume of 5 μl was deposited on a bottom chip. A fraction of solution was confined within the “microwell” chambers. LP-EM observations have been performed after successful leak check of the assembled LC EM-holder (Protochips’ Poseidon).

LP-EM investigations were performed on a double C_s_-corrected JEOL JEM-ARM200F operated at 200 kV and equipped with a cold-field emission gun (Emission current set to 5 μA). Image acquisition in LP-TEM was done on Gatan's Oneview camera. Consecutive series of images (videos) have been acquired applying “*in situ* tools” within Gatan's Microscopy Suite (GMS) version 3.43. “*In situ* Editor” toolbox in the same software was used for post treatments. In LP-STEM, the sample was scanned by a probe with a convergence semi-angle of approximately 23 mrad. ADF-STEM images were collected with an angular collection semi angle range from 33 mrad to 125 mrad.

After, *in situ* investigations in the microscope, LCs were opened and samples were dried at air enabling *ex situ* investigations. For *ex situ* studies, E-chips have been re-assembled in the LC EM-holder with complementary (bottom or top) E-chips without SiN membrane in order to minimize thickness of the samples which causes scattering of the electron beam. EDS signal was collected on a high-angle silicon drift Energy Dispersive X-ray detector (solid angle up to 0.98 steradians with a detection area of 100 mm^2^).

## Conflicts of interest

There are no conflicts to declare.

## Supplementary Material

NR-015-D3NR02400H-s001
