# Supplementary material for: Non-classical crystallization of CeO2 by means of in situ electron microscopy
Source: Nanoscale. 2023 Aug 23;15(35):14595–605. doi: 10.1039/d3nr02400h (PMC10500627; doi:10.1039/d3nr02400h)
Supplement: NR-015-D3NR02400H-s001 [file NR-015-D3NR02400H-s001.pdf]

Electronic Supplementary Material (ESI) for Nanoscale.
This journal is © The Royal Society of Chemistry 2023

Supporting Information: Non-classical crystallization of CeO_2 by means of *in situ* electron microscopy

Hannes Zschiesche[ⱡ]*, *Inna L. Soroka*[§], *Mats Jonsson*[§], *Nadezda V. Tarakina*[ⱡ]*

[ⱡ] Max Planck Institute of Colloids and Interfaces, Department of Colloid Chemistry, Potsdam, Germany.

[§] Applied Physical Chemistry, School of Engineering Sciences in Chemistry, Biotechnology and Health, KTH Royal Institute of Technology, Stockholm, Sweden.

Contact (e-mail) corresponding authors: Hannes.Zschiesche@mpikg.mpg.de (*Hannes Zschiesche*) Nadja.Tarakina@mpikg.mpg.de (*Nadezda V. Tarakina*).

DOSE RATE ESTIMATION

The nominal dose rate in liquid phase transmission electron microscopy (LP-TEM) (cylindrical irradiated volume approximated, in experiment no perfect parallel beam, but slightly divergent leading to a capped cone) was estimated following Eq. 7.8 in [1]:

$$D = 10^5 \cdot Stopping\ power\left[MeV \cdot \frac{cm^2}{g}\right] \cdot \frac{I_{beam}\left[\frac{C}{s}\right]}{A_{beam}[m^2]}$$

(1)

using a total stopping power (density normalized, collisional + radiative) taken from [2] ("water liquid") with an acceleration voltage of the electrons of *200 kV* (thus, *200 keV* energy)

$$S = 2.789\ MeV \cdot cm^2/g.$$

The beam current applied in the experiment was measured using of the calibrated fluorescence screen, when no sample was inserted, to:

$$I_{TEM,beam} = 188.3 \cdot 10^{-12}\ C/s = 117.5 \cdot 10^7\ electrons/s.$$

The area of the beam in LP-TEM was determined on the calibrated pixelated camera for three different dose rate settings:

$$A_{TEM,beam,} = 3.1 \cdot 10^{-12}\ m^2$$

For that given beam area, the dose rate is estimated to:

$$D_{TEM} = 16.9 \cdot 10^6\ Gy/s$$

or in electrons per area per time (electron flux instead of dose rate)

$$D_{TEM} = 3.737\ electrons/A^2/s$$

The dose rate in LP-TEM was set by changing the "C3 condenser lens" current and thereby changing the convergence angle of the electron beam with respect to the sample surface (slightly more parallel for higher dose rate settings), thus, changing effectively the exposed liquid volume while the electron current is kept constant. Even though the approximation of the exposed volume as a cylindrical volume might not hold for a slightly converged beam, it is believed to be a good approximation when the sample is thin and the applied convergence little in comparison to the beam diameter.

In LP- scanning (S)TEM, the electron current of the beam was kept constant as well (gun and condenser settings) and measured on the calibrated (calibrated on both, electron counts and spatially) pixelated camera

$$I_{STEM,Beam} = 1.7 \cdot 10^7\ electrons/s = 2.72 \cdot 10^{-12}\ C/s.$$

A scanning electron beam induces an inhomogeneous electron current density within the scanned region, because the actual irradiation is local (beam on a scanning spot/step or not) and therefore time dependent. That is why the estimated dose rate can be defined in different ways. Either, the dose rate represents an average value over the entire scanned region (represented as an image, each scan spot/step refers to one pixel in the image where the intensity in a specific scattering range was acquired for a dwell time of the beam on those scan spots/steps), similar to the estimation in LP-TEM, here neglecting inhomogeneity caused by the scanning.

$$A_{STEM,expo,1} = 1793.3nm \cdot 1793.3nm = 3\ 215\ 924\ nm^2 = 3.2 \cdot 10^{-12}\ m^2$$

$$A_{STEM,expo,2} = 358.65nm \cdot 358.65nm = 128\ 629\ nm^2 = 1.3 \cdot 10^{-13}\ m^2$$

$$D_{STEM,1} = 2.4 \cdot 10^5\ Gy/s = 0.05\ electrons/s/A^2.$$

$$D_{STEM,2} = 5.9 \cdot 10^6\ Gy/s = 1.32\ electrons/s/A^2$$

This area and time averaged value is within the range of dose rates applied in LP-TEM. A local, temporary dose rate is considered when only the sample volume exposed during a scan step is considered, thus the beam size (e.g. assuming a cylinder volume of *1 nm* in diameter)

$$A_{STEM,beam} = 0.8 \cdot 10^{-18}\ m^2$$

Applying equation (1) with that projected beam area gives a dose rate of

$$D_{STEM,beam} = 9.5 \cdot 10^{11}\ Gy/s = 2.125 \cdot 10^5\ electrons/s/A^2.$$

This value is significantly higher than the dose rate calculated for applied LP-TEM or the dose rate averaged over the entire scanned region. However, it needs to be considered that this dose rate locally applied only for the dwell time of a single scan step ($t_{dwell} = 32\ \mu s$ or less).

QUANTIFICATION OF PROJECTED GROWTH OBSERVATIONS IN *IN SITU* LP-TEM

For a comparable quantification of projected growth, measured objects (primary particles or aggregates) identified in the image have been manually assigned as ovals in Gatan's Microscopy Suite. The two main axes of the ovals have been measured automatically and the average is taken as the determined average diameter, which thereby underlies a coarse approximation as a sphere. Uncertainties are estimated from the standard deviation of the measurements in an image and represent intrinsically the distribution of sizes to some extent.

The higher spatial resolution in the second liquid-cell (LC) (figure 2) enables the determination of primary particle size evolution (orange curve figure S1). This may be possible thanks to a significant thinner liquid sample. A thinner liquid sample would be in agreement with a lower density of formed aggregates (only *3* after more than *4 min* and *30 s* in figure 2 versus more than *30* in the first LC for the same irradiation time in figure 1). No electron energy loss spectroscopy (EELS) measurements are available confirming the relative thickness between both samples.

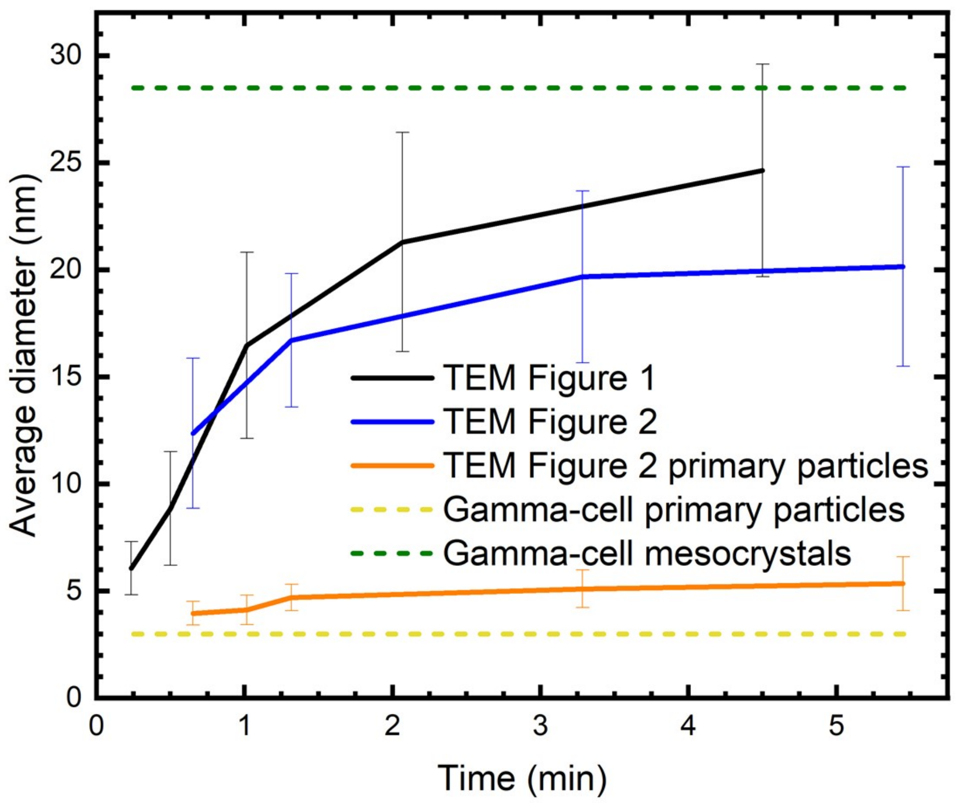

Figure S1. The average diameter of aggregates as a function of time identified in figure 1 and figure 2 (LP-TEM, dose rate $16.7 \cdot 10^{6}$ *Gy/s*). Black and blue curves represent average diameter sizes of aggregates while the orange curve represents the average size of primary particles identified in figure 2. For comparison, maximum measured average diameters of mesocrystals (olive green) and primary particles (yellow) are drawn as horizontal dashed lines.

EX SITU STRUCTURE INVESTIGATIONS

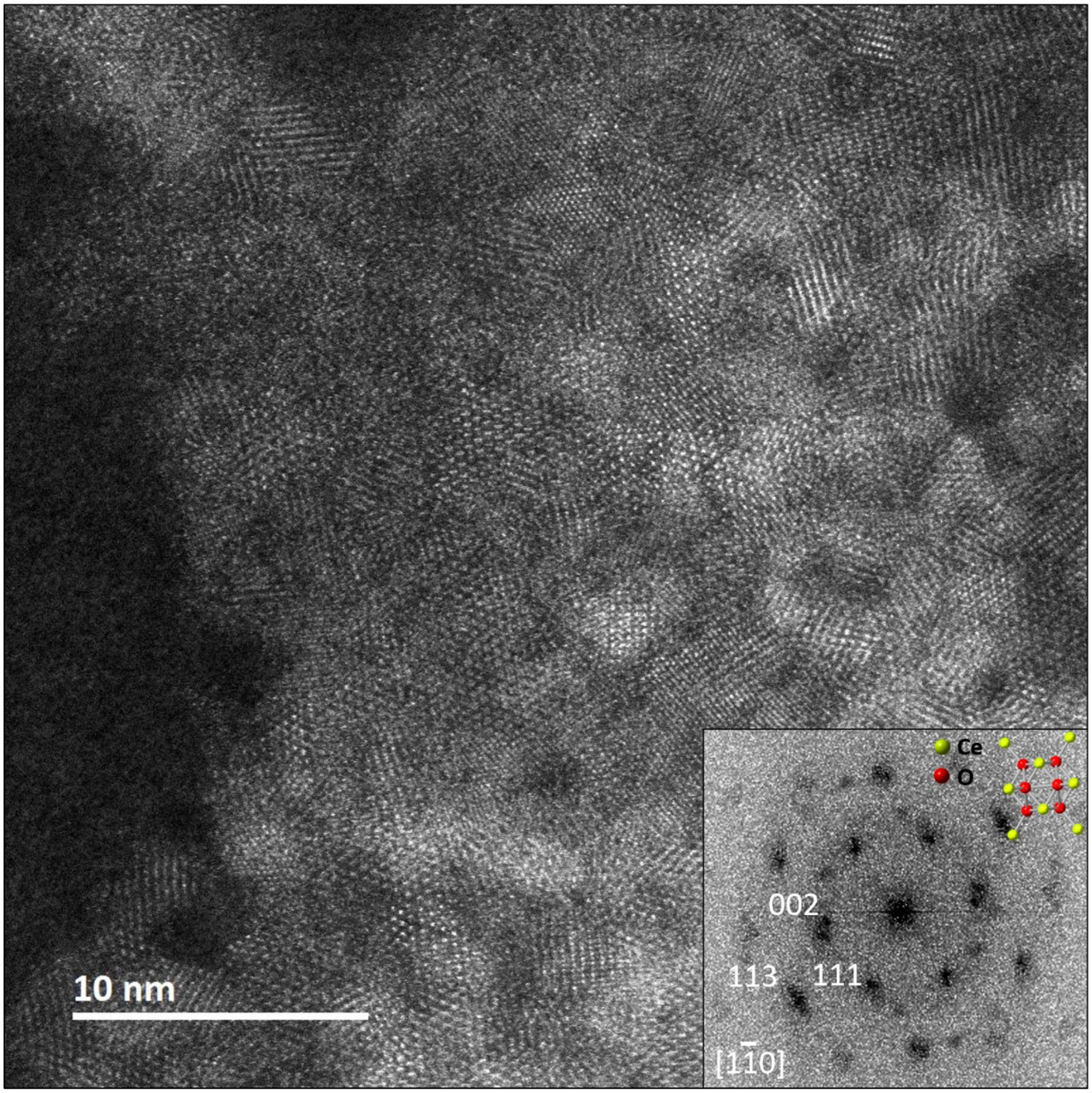

Figure S2. Atomic resolution annular dark-field (ADF)-STEM image of CeO_2 aggregate grown in LP-TEM, dose rate *16.7 ·10⁶ Gy/s*. The microstructure composed of primary particles, which are mutually aligned, is clearly visible. Inset shows the Fourier transformation from the image, it is indexed in [-1-10] zone axis of space group 225 Fm3m [3].

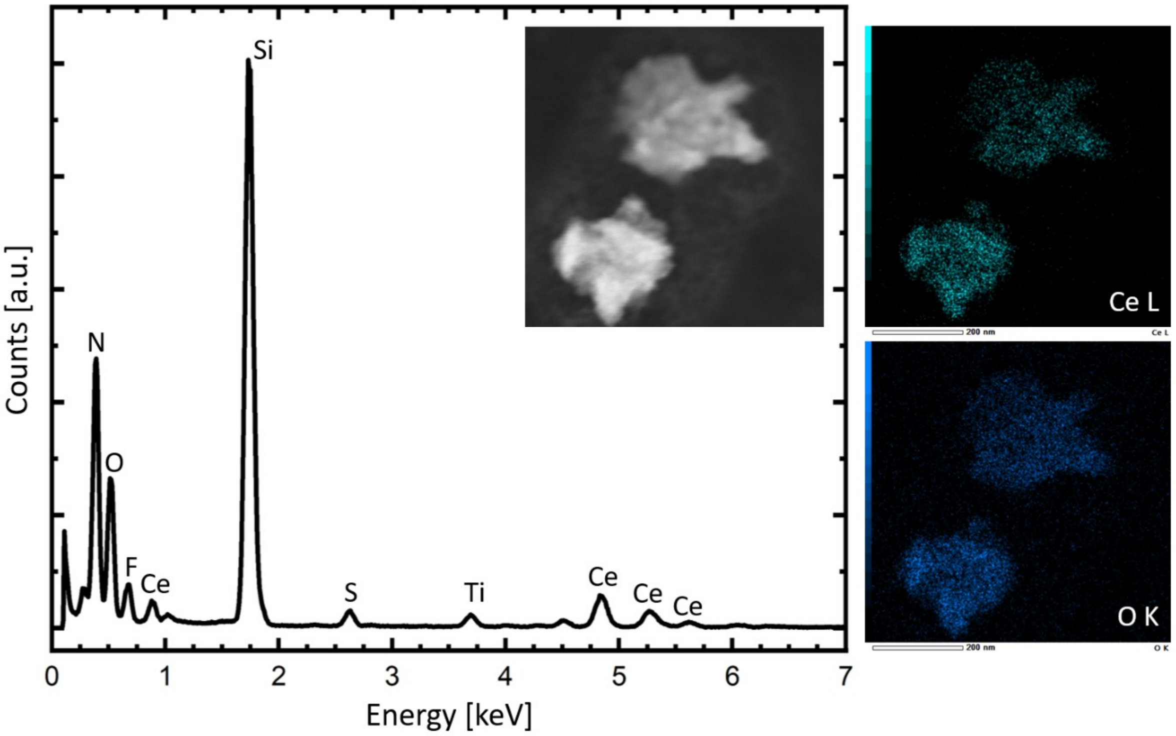

Figure S3. STEM- energy dispersive spectroscopy (EDS) spectrum of the region shown in inset and maps of cerium and oxygen signals.

COMPARISON OF QUANTIFICATION OF PROJECTED GROWTH OBSERVATIONS

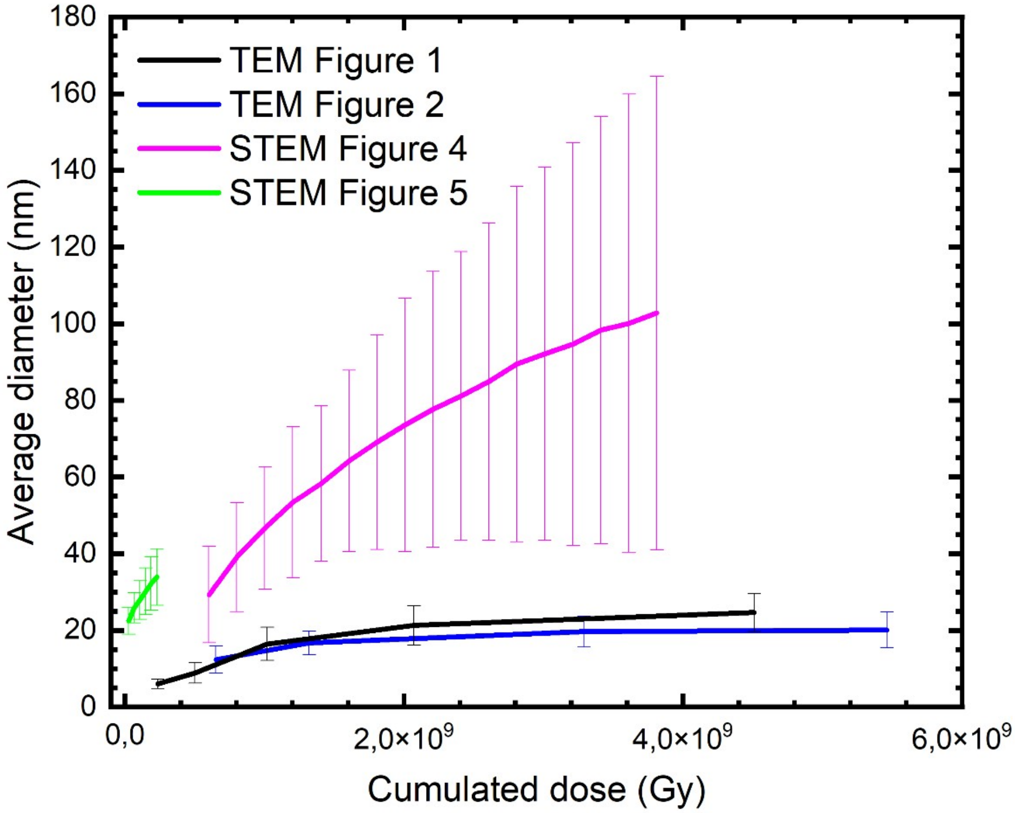

Figure S4. The average diameter of aggregates for different growth modes as a function of cumulated dose.

QUANTIFICATION OF PROJECTED GROWTH OBSERVATIONS IN *IN SITU* LP- STEM

The same method as described above for investigations in *in situ* LP-TEM is applied for investigations in *in situ* LP-STEM.

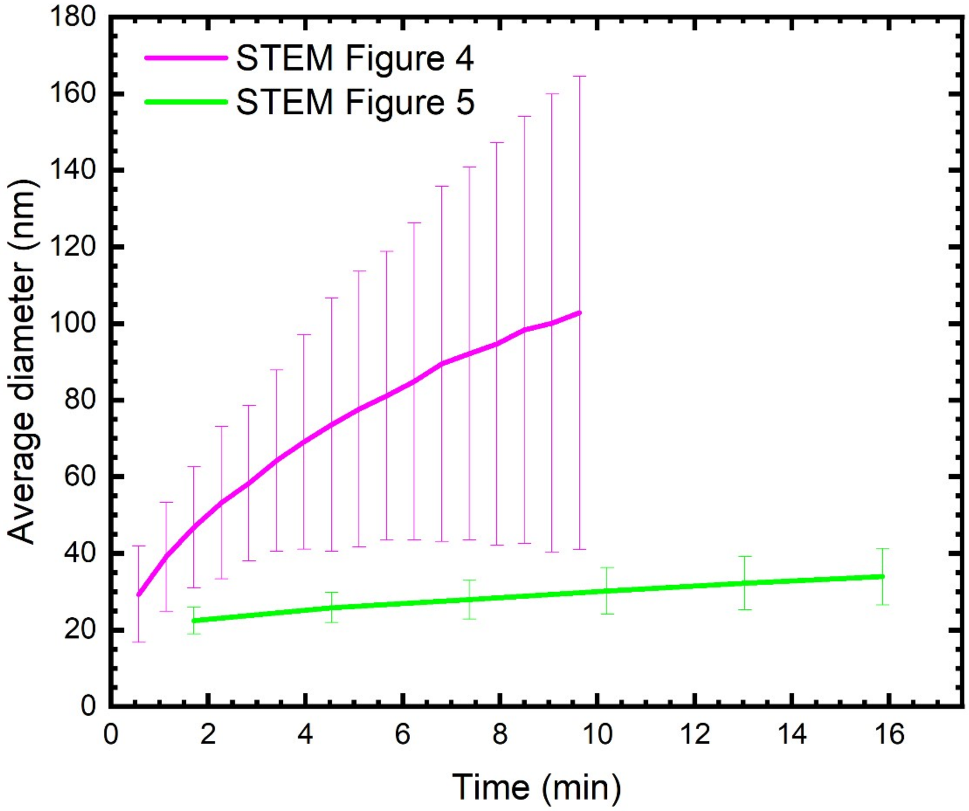

Figure S5. Evolution of the average diameter of aggregates in figure 5 (LP-STEM, average dose rate of $5.9 \cdot 10^6$ *Gy/s*) and in figure 5 (LP-STEM, average dose rate $2.4 \cdot 10^5$ *Gy/s*). Note that *34 s* refer approximately to one scan frame.

ZOOM OUT FROM REGION OF INTEREST IN LP-STEM

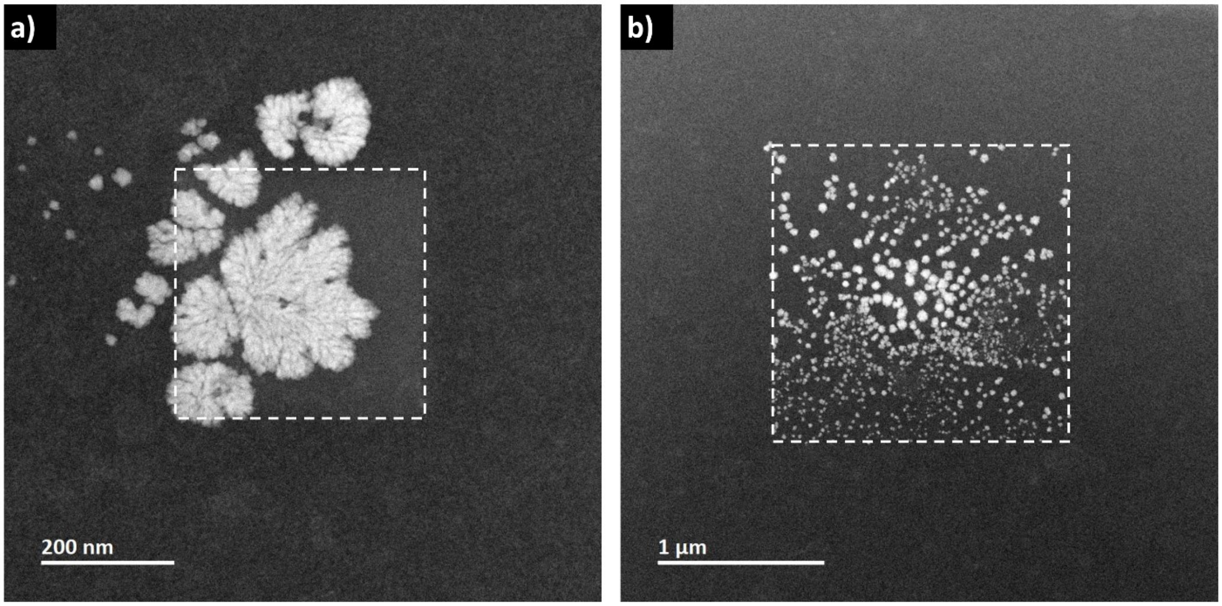

Figure S6. Overview ADF-STEM images taken after *in situ* LP-STEM investigations described in the main text. a) High average dose rate investigation (figure 5). Branched structures are formed even outside the irradiated liquid volume. b) Lower average dose rate investigation (figure 6). No crystals formed outside the irradiated liquid volume or at its rim.

References

[1] F. M. Ross, Liquid Cell Electron Microscopy, New York: Cambridge University Press, 2016.

[2] , [Online]. Available: https://physics.nist.gov/cgi-bin/Star/etable.pl. [Accessed 13 10 2022].

[3] C. Artini, M. Pani, M. M. Carnasciali, M. T. Buscaglia, J. R. Plaisier and G. A. Costa, "Structural Features of Sm- and Gd-Doped Ceria Studied by Synchrotron Diffraction and micro-Raman Spectroscopy," *Inorganic Chemistry,* vol. 54, pp. 4126-4137, 2015, doi: 10.1021/acs.inorgchem.5b00395.
